# A Micro-Pressure Sensing Method Based on the Micropatterned Electrodes Filled with the Microspheres

**DOI:** 10.3390/ma10121439

**Published:** 2017-12-18

**Authors:** Jianli Cui, Binzhen Zhang, Junping Duan, Hao Guo, Jun Tang

**Affiliations:** Science and Technology on Electronic Test & Measurement Laboratory, North University of China, Taiyuan 030051, Shanxi, China; b1506014@st.nuc.edu.cn (J.C.); dunjunping@nuc.edu.cn (J.D.); tangjun@nuc.edu.cn (J.T.)

**Keywords:** pressure sensor, flexible sensor, micropatterned structure, PS microspheres

## Abstract

As the core component of the sense of touch, flexible pressure sensors are critical to synchronized interactions with the surrounding environment. Here, we introduce a new type of flexible capacitive pressure sensor based on a template of electrodes, with a one-dimensional pyramid micropatterned structure on a Polydimethylsiloxane (PDMS) substrate and a dielectric layer of polystyrene (PS) microspheres. The proposed sensor exhibits a stable and high sensing sensitivity of 0.741 kPa^−1^ to capacitance, good durability over 1000 cycles, and fast response time (<150 ms). Our flexible capacitive sensor responds not only to pressure but also to bending forces. Our device can be used to monitor the location and distribution of weight pressure. The proposed capacitive pressure sensor has itself been applied foreground in lots of aspects, such as electronic skins, wearable robotics, and biomedical devices.

## 1. Introduction

Flexible pressure sensors have been well-documented, owing to their new and untraditional applications in robotic systems, electronic skin, touch interfaces, and wearable medical devices. Researchers have continued to focus on improving the sensing sensitivity and reliability of flexible pressure sensors for application in a pressure range of less than 50 kPa to mimic human skin and tactile receptors. Until now, many methods have been proposed to realize pressure sensitivity based on piezoelectric [1,2,3], piezoresistive [4,5], and piezocapacitive types [6,7] of sensors. Among them, the capacitive type has been most widely investigated, due to their advantages when compared to the other two types: a low power consumption, low sensitivity to humidity and temperature, and highly repeatable response [8,9].

Various transduction methods and structures for flexible capacitive pressure sensors have been presented in the literature in the past few years. Capacitive-type pressure sensors that are based on elastomeric dielectric materials have also been widely demonstrated [10,11,12]. When an external pressure is loaded on the sensor, the elastomeric dielectric layer exhibits different deformation, which leads to a variation in the capacitance. Nanomaterials, such as nanowires [13,14,15], carbon nanotubes [4,16], polymer nanofibers [5,17], metal nanoparticles [18], and graphene [19] have been used with elastomeric dielectric layers to develop novel flexible capacitive pressure sensors. In addition, researchers have used some metals (Au film, Ag film, Ag nanowire networks) or liquid metal as the top and bottom electrode materials of capacitive pressure sensor devices. These sensors exhibit mechanical stretchability, however, they are limited by their low pressure sensitivity. To improve the properties of capacitive-type sensors, researchers have proposed using microstructures [20,21] in the elastomeric dielectric layer. Modifying the devices in this manner has produced pressure sensors with high pressure sensitivity and good flexibility [19,22]. Kim et al. [22] reported a highly sensitive capacitive pressure sensor based on a polymer dielectric film with a nano-needle structure. However, the many associated drawbacks should be taken into account, such as manufacturing difficulty of sophisticated micro/nanostructures, demanding materials, expensive cost, challenging scalability, and poor adhesiveness between the substrate and metal layer.

Here, we report a novel flexible capacitive pressure sensor based on fabricating electrodes with a pyramid structure on a polydimethylsiloxane (PDMS) substrate through the microelectromechanical system (MEMS) process. The device was designed based on a template for electrodes with a micropatterned one-dimensional pyramid structure on a PDMS substrate and a dielectric layer of polystyrene (PS) microspheres. To intensify the adhesion between the PDMS substrate and metal materials, the PDMS surface was modified with O_2_ plasma, combined with a sodium dodecylian sulfate (SDS) solution. Observations showed that the sensor exhibited high sensitivity, fast response to bending, and great durability and repeatability. The sensor was able to effectively monitor the distribution and the position of the external loading. Our work provides a new method for wafer-level fabrication of flexible electronic devices based on MEMS technology. 

## 2. Experimental Details

### 2.1. Fabrication of Flexible Micropatterned Electrodes on PDMS Substrates

**Figure 1:**
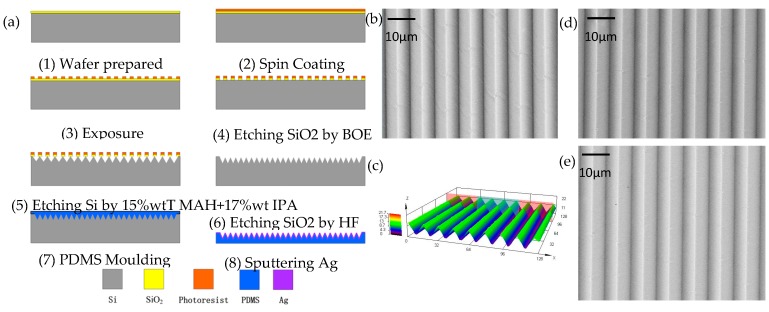
Fabrication process and morphology characterization of the micropatterned electrodes on the Polydimethylsiloxane (PDMS) substrates. (**a**) Fabrication process of the PDMS electrode with the Ag-embedded pyramid structure; (**b**) scanning electron microscopy (SEM) image of the electrode with a one-dimensional pyramid structure showing high uniformity; (**c**) Laser confocal image of the electrode pattern; (**d**,**e**) SEM image of the micropatterned electrodes on the PDMS substrates after replication from the same Si mold.

Silver was used as the electrode material [8,23,24]. Figure 1a shows the manufacturing process of the flexible Ag electrodes on the PDMS substrate. The microelectrode that is described in this paper was fabricated using the MEMS process [25]. This process consists of six steps, is relatively simple, and it has great potential for application to wafer-level volume production. First, a positive photoresist (S1805) was spun-coated on a 4-inch silica wafer at 3000 rad/min and soft-baked at 105 °C for 90 s. Second, the wafer was exposed to a dose of 135 mJ/cm^2^ with a mask aligner (EVG-610, Eastern Venture Group Co., Ltd., Kanagawa, Japan). Post-exposure baking was performed at 115 °C for 120 s to form strong crosslinks. After the wafer had gradually cooled down, the structure was immersed in a positive developer (40 s). Third, a corrosion experiment was carried out involving two steps: etching SiO_2_ using a buffered oxide etch and etching Si using 15 wt % TMAH + 17 vol % isopropyl alcohol (22 min). Fourth, a SiO_2_ layer was removed with hydrofluoric acid. Then, a micropatterned structure looking like continuous one-dimensional pyramid on the Si mold was obtained, as shown in Figure 1(a6). Fifth, a PDMS mold was prepared by mixing the liquid PDMS elastomer (Sylgard 184, Dow Corning, Midland, MI, USA) and a curing agent at a 10:1 ratio by volume. The liquid mixture was poured onto the Si mold and was thermally cured at 75 °C for 2 h to obtain a micropatterned structure on the PDMS substrate. The cured PDMS was disposed (120 W, 20 s) by plasma (ION40) to reinforce surface adhesion, and then immersed in a Sodium Lauryl Sulphate (SDS, Shengtongxin Company, Tianjin, China) solution (0.5%, 15 s) to form –SO_3_^−^ groups on the surface of the PDMS substrate with the pyramid structure. This process can introduce a condensation reaction of hydrophilic functionalities, and lead to close contact between PDMS and Ag^+^ [21,26,27]. Finally, micropatterned electrodes on the PDMS substrates were obtained through a magnetron sputtering operational (60 W, 8.0 × 10^−3^ Torr) to coat the surface of the PDMS mold with an Ag layer. In contrast, we used the same fabrication method, as described above, to obtain nonpatterned stretchable electrodes, except that the PDMS prepolymer was coated onto a slippery substrate.

### 2.2. Design and Fabrication of Flexible Pressure Sensors 

**Figure 2:**
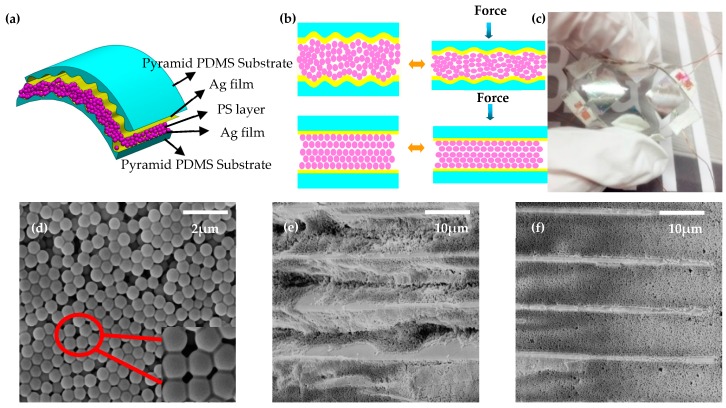
Schematic of the flexible capacitive pressure sensor and SEM images of polystyrene (PS) microspheres. (**a**) Schematic of the capacitive pressure sensor; (**b**) Deformation of the pressure sensors with and without one-dimensional pyramid structure PDMS electrodes; (**c**) Pressure sensor array with 2 × 2 sensing element; (**d**) SEM image of PS microspheres at low and high amplification; Morphology and displacement changes of the flexible sensor (**e**) before and (**f**) after compression caused by application of external pressure.

Figure 2a shows the schematic design of the pressure sensor with the micropatterned electrodes based on the principle of the capacitive sensor. The device was composed of the bottom and top parallel-plate micropatterned electrodes on the PDMS substrate, and a dielectric layer of PS microspheres. The fabrication process contains the following steps: First, the top and bottom Ag electrodes were fabricated via sputtering of 400 nm thick Ag films. During this process, a metal mask was placed on the PDMS substrate with the pyramid structure to form two parallel electrodes with dimensions of 0.9 (length) × 0.9 (width) cm^2^. The metallic electrodes were deposited in a high vacuum system that was equipped with a DC magnetron sputtering source (QPrep400, Mantis, Toronto, UK) under the following conditions: DC power of 120 w; Ar flow rate of 30 sccm, chamber pressure of 7.5 × 10^−3^ Torr, and sputtering time of 5 min. Then, the PS microspheres were spin-coated upon the prepared top and bottom Ag miropatterned electrodes to form the middle dielectric layer. Finally, the top and bottom electrodes with a pyramid structure of the sensor were aligned face to face and bonded through the PDMS self-assembling ability. The fabrication process of the non-patterned sensor was nearly the same, except that non-patterned PDMS substrates was used. To facilitate comparison, the pressure sensors were designed with uniform dimensions of 0.9 × 0.9 cm^2^, and the quantity of PS microspheres was consistent.

## 3. Results and Discussion

Through scanning electron microscopy (SEM, Hitachi, Tokyo, Japan) characterization, a regular one-dimensional pyramid structure on the PDMS substrate with a periodicity of 12 μm and height of about 9 μm were achieved by duplicating microstructures from the Si template that was obtained with the above MEMS process, as it is shown in Figure 1c. In addition, one template can be reutilized many times, and many more films were reproduced, as shown in Figure 1e,f. The results showed that the manufactured films were mostly similar in the structure, quantity, and morphology of the replicated pyramid structures. Thus, the repeated action did not influence the consistency of the micropatterned structures on the PDMS substrates.

Figure 2a shows a schematic diagram of the capacitive pressure sensor based on electrodes with one-dimensional pyramid structures on the PDMS substrate. Figure 2b shows a physical photo of the 2 × 2 sensor array, where each sensing unit has dimensions of 0.9 × 0.9 cm^2^. In contrast to the traditional capacitive pressure sensor, this device was fabricated on the basis of parallel-plate capacitor principle and is composed of two parts. Two Ag-embedded electrodes with a pyramid structure on the PDMS substrate were used as parallel-plate electrodes, and PS microsphere were used as the middle dielectric layer. As illustrated in Figure 2d, SEM images indicated a high uniformity of the PS microspheres, which had an average diameter of 550 nm. This dimension is far less than that of pyramid structure on the PDMS substrate. Thus, the PS microspheres tended to fill the depressions of the pyramid during spin-coating. The PS spheres were distributed one by one between the one-dimensional pyramid microstructures of two capacitance plates. Under the pressure, the PS spheres would be against the one-dimensional pyramid microstructures and applied the pressure on it, which can cause the one-dimensional pyramid microstructures micro-deformation. The micro-deformation of millions of the one-dimensional pyramid microstructures would bulk up, and then generate a lager deformation on the capacitance plates. It is the reason to make the capacitance change, contribute to improve the sensitivity. Figure 2e,f exhibits the morphological and displacement change with an external pressure load. They indicate that the PS spheres are packed into the interspace that is normally occupied by air with the pressure load.

**Figure 3:**
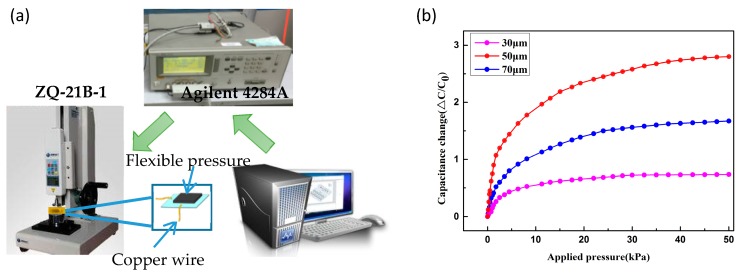
(**a**)Test system construction; (**b**) The effect of various PS thicknesses to the sensitivity of the sensors.

Figure 3a shows the experimental test setup that is used to characterize the capacitive pressure sensor. A compression-testing machine (ZQ-21B-1, Zhiqu Precision Instrument Co., Ltd., Dongguan, China) was used for force application and measurement. The sensor output, i.e., capacitances, was measured by an impedance analyzer (4284A, Agilent, Santa Clara, CA, USA). During the measurement process, a copper wire was served as a connecting link between the sensor and the capacitance meter to improve the measurement stability of the device. A variable external pressure of 0–50 kPa was loaded, and the capacitance of 3.522 pF at a load of 0 Pa was used to represent the initial capacitance *C*_0_.

**Figure 4:**
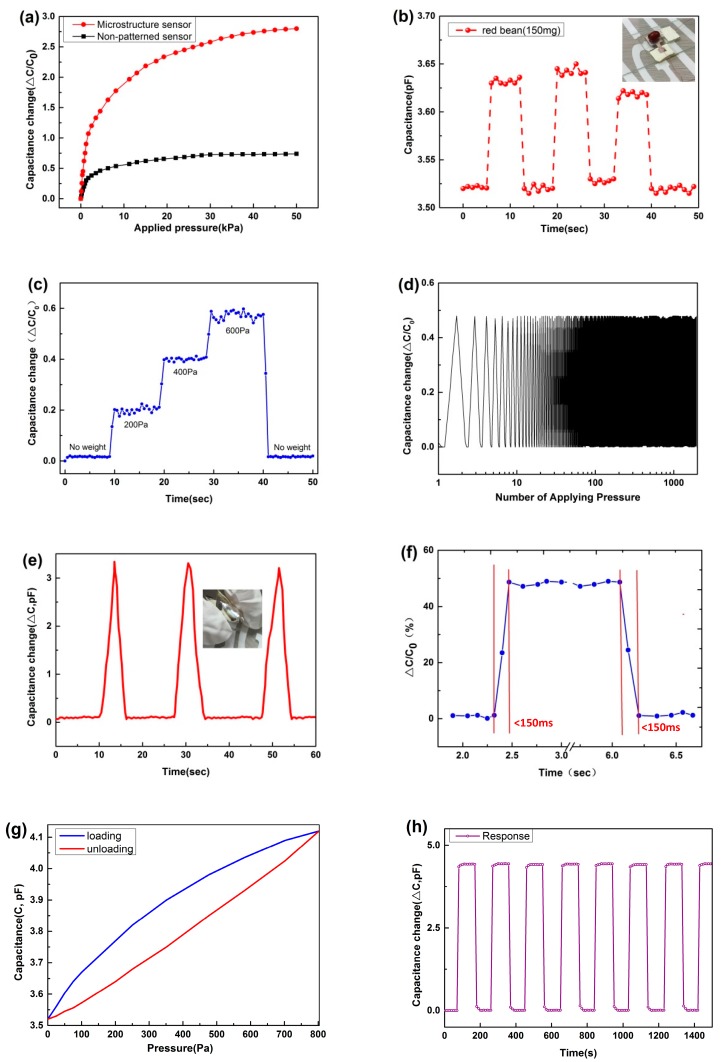
Pressure response capabilities of the capacitive pressure sensor. (**a**) Relative capacitance change-pressure curve of the microstructure capacitance sensor (red) and non-structure sensor (black); (**b**) Capacitance–time curve with loading and unloading of a red bean (corresponding pressure of 19 Pa); (**c**) Real-time monitoring for applied pressure of 0.2, 0.4 and 0.6 kPa; (**d**) Stability test of the sensor wirh loading/unloading pressure over 1000 cycles; (**e**) Bending force (about 120°) was loaded onto the flexible pressure sensor; (**f**) Fast response and relaxation time (<150 ms) of the pressure sensor; (**g**) Hysteresis of output capacitance signal for an input pressure of ~800 Pa with 1 Hz force frequency; and, (**h**) Capacitance response curve of the microstructured sensor versus time over 10 pressing/releasing cycles with a pressure of 20 kPa.

The capacitance of a parallel-plate capacitor can be defined as *C* = (*ε · S*)/*d*, where ε represents the dielectric constant, S denotes the relative area plate, and d is the plate distance. In order to further understand the sensing performance of sensors with and without the micropatterned electrodes, the electric characteristics of the two types of sensors were investigated under different pressure loads, as shown in Figure 4a. The sensitivity can be expressed as *S =* (*ΔC*/*C*_0_)/*P*, where *ΔC* denotes the change of capacitance (*C*–*C*_0_) and *P* is the pressure load. *P* can be calculated as *F*/*A,* where *F* is the force that is loaded on the surface of the sensing unit and *A* is the area of the parallel-plate electrode [28]. As illustrated in Figure 4a, the piezocapacitive sensor exhibited two linear regions in the relationship between the loaded pressure and sensitivity. For the microstructured sensor, the first linear region exhibited a high pressure sensitivity of 0.741 kPa^−1^ in the low pressure range (0–1 kPa). This excellent pressure-sensing capability can be attributed to three factors. When pressure was applied to the sensors, the unique geometry of the one-dimensional pyramid structure that was resting on flexible PDMS substrates was extended, which expanded the relative plate area. At the same time, the decreased distance that was induced by the pressure caused a high variation in the capacitance (*ΔC*) of the sensor. Moreover, the PS spheres would be against the one-dimensional pyramid microstructures and applied the pressure on it, which can cause the one-dimensional pyramid microstructures micro-deformation. The micro-deformation of millions of the one-dimensional pyramid microstructures would bulk up, and generate a lager deformation on the capacitance plates. It is the reason to make the capacitance change, contribute to improve the sensitivity. In addition, a little air in the gap was left during bonding process of the two substrates. Under the pressure, the air in the gap would be compressed to increase the dielectric properties, which contribute significantly to improve the sensitivity. The sensitivity slightly decreased as the applied pressure increased; however, the sensitivity of 0.010 kPa^−1^ is comparable to that reported by previous groups [29]. The non-microstructure sensor demonstrates a lower sensitivity of 0.246 kPa^–1^ in the low pressure range, which decreased to 0.0005 kPa^−1^ as the applied pressure was increased. To investigate how the amount of PS spheres affect the sensitivity, the various PS spheres thicknesses with 30, 50, and 70 μm were spin-coated on the surface of electrodes with one-dimensional pyramid structures on the PDMS substrate, and the sensitivity results are shown in Figure 3b. When the PS spheres thickness is 50 μm, the results demonstrated their ultrasensitive properties. The sensor exhibits small capacitance variation until the PS spheres thickness is up to 70 μm. The reason is that the thickness of the PS spheres corresponds to the thickness of the dielectric layer, as well as the distance between the two plates. Small deformation of the electrode surface has a little effect on capacitance variation of the sensor with a thick PS spheres when a pressure was applied to the sensors. In contrast, the sensor with ultrathin dielectric layer presents a limited deformation during the whole compression process.

As illustrated in Figure 4b, we investigated the rapid response capability and reproducibility of the flexible sensor by loading and unloading a red bean with a low weight of 150 mg, corresponding to a pressure of 19 Pa. When the red bean was loaded, the capacitance changed from about 3.522 to 3.634 pF. After the red bean was removed, then the capacitance value returned to around 3.522 pF. This confirmed that the sensor had good stability and rapid response capability. When a ladder-like rising and falling pressure load was applied (200 → 400 → 600 Pa → no load), as shown in Figure 4c, the sensor presents a rapid response time of less than 500 ms. When the external loading was removed, then the capacitance presented the original value, regardless of the previous pressure. This indicates that the sensor can be used to real-time pressure monitoring.

As presented in Figure 4d, a repeatable and reliable response with no evident hysteresis was observed over 1000 loading–unloading cycles. The capacitance amplitude exhibited insignificant changes for the whole cyclic curve. Moreover, the pressure sensor demonstrated good flexibility, which was attributed to the highly flexible micropatterned PDMS/Ag electrodes. Further, it could be bent randomly in every direction. Figure 4e shows the real-time *∆C*–*t* curve when the sensor was repeatedly bent (about 120°, holding for 10 s) a few times. The device showed an apparent pattern in its response to the repeated bending forces. As shown in Figure 4f, we also investigated the response time of the device through loading and unloading an abrupt pressure. The results showed that the response and relaxation times were each less than 150 ms. To understand the robustness of the flexible pressure sensor to compression cycles, the hysteresis of output capacitance change for an input pressure of ~800 Pa with 1 Hz force frequency have been indicated, as illustrated in Figure 4g. This hysteresis may be attributed to elastic displacement of PS spheres during unloading and the viscoelastic effects of the PDMS substrate layers. To better understand the performance of the sensor with micropatterned electrodes that are filled with microspheres, we tested the sensor performance with pressure loading and unloading. Figure 4h shows the change in capacitance of the sensor with micropatterned electrodes versus time with repeated pressing/releasing cycles of a pressure of 20 kPa. The configuration of the curve is very smooth and shows that our device exhibited excellent stability and repeatability.

**Figure 5:**
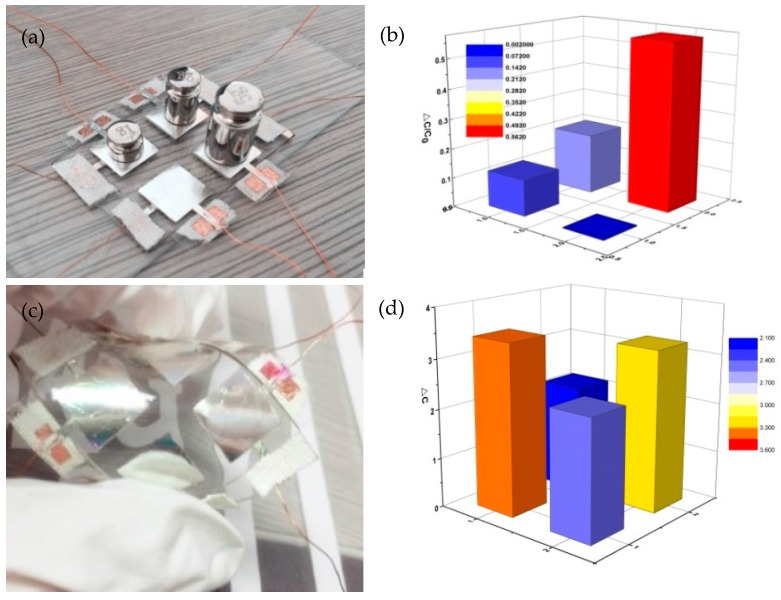
Property characterization of the pressure sensor array. (**a**) Picture of the 1, 2, and 5 g weights placed on the 2 × 2 sensor array; (**b**) Pressure-mapping capability of the sensor array; (**c**) Picture of a bending (about 120°) applied to the sensor array; and (**d**) Pressure-mapping capability of the sensor array.

To demonstrate the efficiency of this sensor, we explored a pressure sensor array with four pixels, in which each pixel can be considered as a chip-type sensing component with dimensions of 9 × 9 mm^2^, as shown in Figure 5a. The device monitored the weight distribution on each component by measuring the capacitance variation. For demonstration purposes, different weights (1, 2, and 5 g) were placed on the surface of the sensor array, as shown in Figure 5a,b presents the corresponding result on a capacitance map. Only the capacitances of the components with weights increased observably. The capacitance change of the component with a 2 g weight was greater than that of the component with a 1 g weight, and the changes for the component with a 5 g weight were higher than those for the first two. In order to further understand the bending performance of the sensor with the micropatterned electrodes that were filled with microspheres, the sensor was bent (about 120°) a few times, as illustrated in Figure 5c. Each sensing component exhibited a different bending camber, respectively. Figure 5d demonstrates the corresponding capacitance change in the sensor array. The capacitance map illustrates that our device can detect different bending forces. The experimental results show that the manufactured sensor array can not only concurrently and accurately monitor various weight pressures placed on its surface and identify the local pressure distributions, but also measure the applied bending force. These properties are typical of wearable devices.

## 4. Conclusions

A novel flexible pressure sensor based on polystyrene microspheres and an electrode with a micropatterned pyramid structure on a PDMS substrate was successfully demonstrated. The electrodes with a one-dimensional pyramid structure were fabricated by using MEMS technology. The available sensor presented a high sensitivity of 0.741 kPa^−1^, good repeatability over 1000 cycles, and a fast responses time of <150 ms. The novel flexible sensor can accurately and flexibly monitor the distribution and position of the external pressure because of its excellent characteristic. Drawbacks, such as bending and deformation during the fabrication process resulting in sensor failure, were effectively solved by the proposed Ag electrodes with a pyramid structure. Our work provides a new method for wafer-level fabrication of flexible electronic devices based on MEMS technology. The flexible sensor can be utilized to monitor both pressure and bending forces, thus, it is especially suitable for future applications of electronic skin in smart robotic systems.
